# A Decade of the Global STI Vaccine Roadmap: Progress, Gaps and Challenges

**DOI:** 10.1002/jia2.70142

**Published:** 2026-07-24

**Authors:** Remco P. H. Peters, Sinead Delany‐Moretlwe, Kate L. Seib, Birgitte Giersing, Sami L. Gottlieb

**Affiliations:** ^1^ World Health Organization Geneva Switzerland; ^2^ Wits RHI, University of the Witwatersrand Johannesburg South Africa; ^3^ Institute for Biomedicine and Glycomics Griffith University Gold Coast Queensland Australia

1

Progress in sexually transmitted infection (STI) control has remained limited over the past decades, reflecting chronic under‐investment, persistent structural and social barriers, high levels of asymptomatic infection and limited integration of STI services within broader healthcare systems [1]. In this context, the development and deployment of effective STI vaccines is essential and recognized by the World Health Organization (WHO) as a global research priority [2].

*The global STI vaccine roadmap* was developed by WHO, the U.S. National Institutes of Health and international partners in 2014 and updated in 2016 [3]. It outlines a coordinated strategy to accelerate the development, evaluation and introduction of new STI vaccines across three domains: assessing the public health value, advancing research and development, and defining preferred attributes and use cases for implementation (Figure 1a). More than a decade later, substantial progress has been made in several key areas, though major gaps and challenges remain.

**FIGURE 1:**
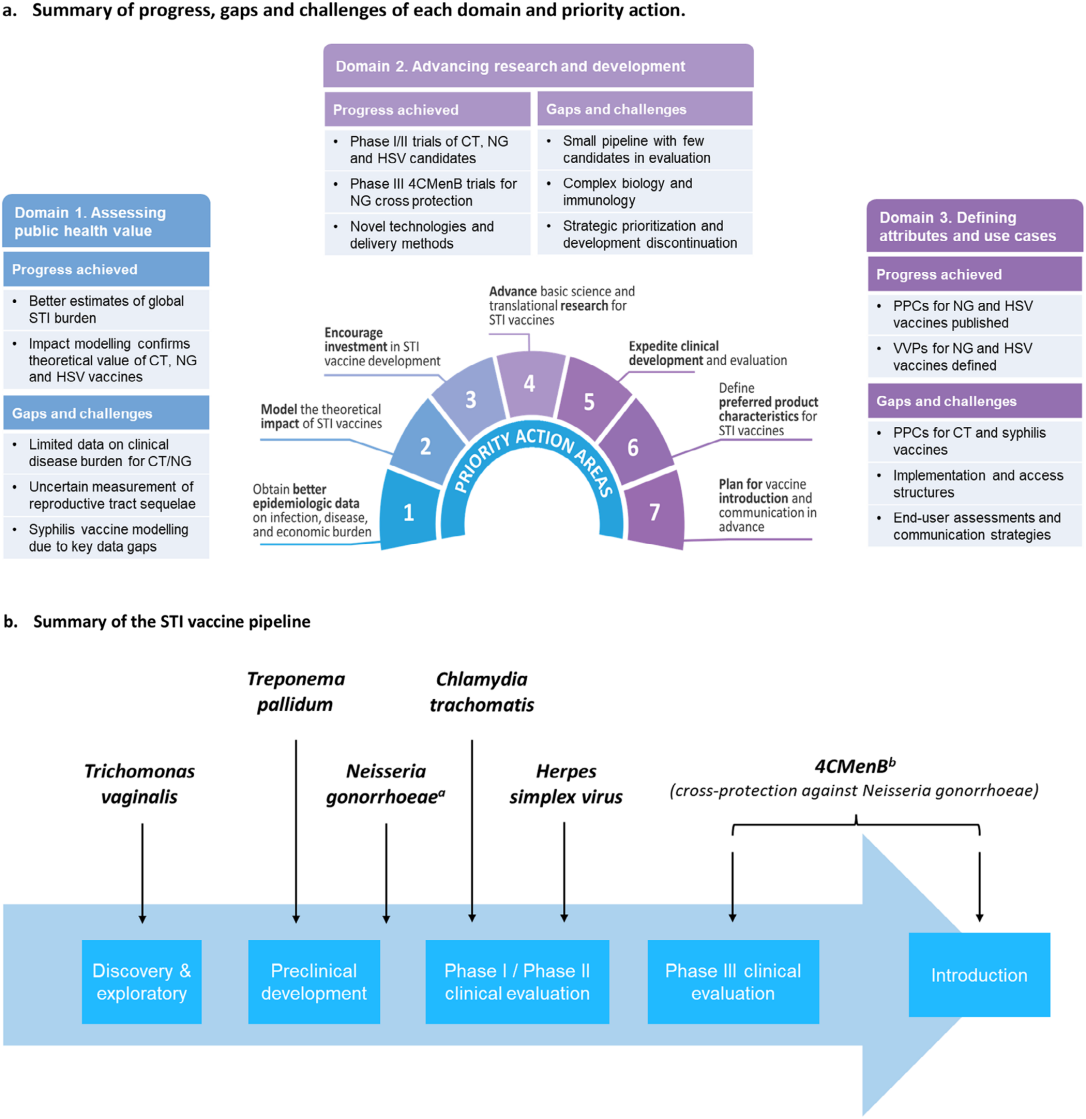
Status of the global STI vaccine roadmap. (a) Summary of progress, gaps and challenges of each domain and priority action. (b) Summary of the STI vaccine pipeline. Abbreviations: CT, *Chlamydia trachomatis*; HSV, herpes simplex virus; NG, *Neisseria gonorrhoeae*; PPCs, preferred product characteristics; STI, sexually transmitted infection; VVP, vaccine value profile. ^a^Results of phase I/II studies of an investigational vaccine against *Neisseria gonorrhoeae* have not yet been reported, but the developer has indicated that the product is removed from the pipeline [12]. ^b^Phase III trials are expected to read‐out in 2026; the United Kingdom has introduced the vaccine based on modelling and observational evidence, which will generate real‐world effectiveness data.

*Public health value assessments* underpin all other efforts to catalyse STI vaccine development. Advances are most marked for HSV vaccines, with updated estimates for global HSV‐2 prevalence (∼520 million people aged 15−49 years), burden of disease (∼188 million cases of genital ulcer disease and 14,000 neonatal herpes cases annually), and economic burden ($35 billion in 2016) [4]. Mathematical modelling suggests that both prophylactic and therapeutic HSV‐2 vaccines could have important population‐level impacts on HSV‐2 incidence and genital ulcer disease, as well as indirect effects in reducing HIV risk [5 ^,^ 6]. In contrast, the public health value assessment for chlamydia and gonorrhoea vaccines is constrained by limited data on disease outcomes. Although annual burdens of chlamydia (129 million new cases) and gonorrhoea (82 million new cases) are high, attribution to clinical disease, reproductive tract complications and healthcare utilization is poorly quantified. Mathematical modelling suggests that even partially protective vaccines (25%–50% vaccine effectiveness) could reduce *N. gonorrhoeae* incidence or prevalence [7 ^,^ 8], and could delay antimicrobial resistance development [9]. Vaccine modelling for syphilis has been limited due to the nascent stage of vaccine development.

*Research and development pathways* for STI vaccines have been dynamic, with several candidates now under clinical evaluation (Figure 1b). Unexpectedly—given the landscape at the time the STI roadmap was developed—the greatest progress has occurred in gonorrhoea vaccine development. The feasibility of *N. gonorrhoeae* vaccines is driven by observational evidence of cross‐protection from outer membrane vesicle (OMV)‐based meningococcal B (MenB) vaccines. Pooled data from observational studies estimate 30%–35% MenB vaccine effectiveness against gonorrhoea [10], and an Australian immunization programme reported 42% protection within 5 years of vaccinating adolescents [11].

Based on modelling and observational evidence, the United Kingdom introduced 4CMenB (Bexsero) vaccination in 2025 in sexual health clinics [12]. However, in the GoGoVax randomized controlled trial conducted among 650 gay and bisexual men in Australia, two doses of 4CMenB did not reduce gonorrhoea incidence compared with placebo (incidence rate ratio 1.01; 95% confidence interval [CI] 0.80−1.26) [13]. This aligned with results of two other trials that showed no statistically significant protection of the 4CMenB vaccine against gonorrhoea, the ANRS 174 DOXYVAC trial (adjusted hazard ratio 0.78, 95% CI 0.60−1.01) and the MenGO trial (incidence rate ratio 0.78, 95% CI 0.40−1.51) [14 ^,^ 15]. Results from ongoing 4CMenB vaccine trials in women and lower‐incidence populations will be critical for future recommendations. These findings are explored further elsewhere in this supplement [16].

Research on 4CMenB has significantly advanced the development of *N. gonorrhoeae*‐specific vaccines by driving progress in immunology, microbiology and assay development. One dedicated vaccine candidate—*N. gonorrhoeae* GMMA—has reached phase I/II evaluation (NCT05630859), but development was discontinued following strategic reprioritization by GSK (www.gsk.com/media/lkklkfgi/q3‐2024‐pipeline‐assets‐and‐clinical‐trials‐report.pdf). Additional candidates are in development, including a native OMV (nOMV) vaccine (https://www.jenner.ac.uk/research/infectious‐diseases/gonococcal‐vaccine‐project) and a multi‐antigen vaccine (https://lmtbio.com/company).

Several prophylactic and therapeutic HSV‐2 vaccine candidates have advanced through the clinical pipeline, but most were discontinued for scientific or strategic reasons [17]. Moderna halted its mRNA‐1608 (NCT06033261) programme in November 2025 due to strategic reprioritization (https://feeds.issuerdirect.com/news‐release.html?newsid=5758345706655596&symbol=MRNA); however, BioNTech's trivalent mRNA prophylactic HSV vaccine candidate (BNT163) continues in active clinical evaluation (NCT05432583). While earlier HSV candidates failed to demonstrate durable clinical efficacy, renewed activity using mRNA, viral vector and next‐generation protein‐subunit platforms reflects ongoing efforts to improve antigen selection, enhance mucosal immune responses and better account for biological challenges. Interactions between HSV‐1 and HSV‐2 immunity complicate vaccine design, as pre‐existing HSV‐1 infection has been associated with reduced efficacy against HSV‐2 for at least one candidate. Efficacy against both viruses will be desirable.

Although *C. trachomatis* is the most common bacterial STI, progress in vaccine development has been modest. Chlamydia vaccine development faces key challenges, including difficulty measuring chlamydia‐associated genital tract morbidity and specific safety considerations, as immune responses have been linked in some animal models to enhanced genital tract inflammation following reacquisition [18]. Two vaccine candidates are currently in clinical evaluation. Phase I studies of CTH522, a recombinant major outer membrane protein–based vaccine candidate, demonstrated a favourable safety profile and induced robust T‐cell and neutralizing antibody responses across multiple *C. trachomatis* serovars [19]. These findings support biological feasibility while underscoring the need for trials powered to evaluate clinical efficacy endpoints. In parallel, Sanofi has initiated early clinical development of an mRNA‐based chlamydial vaccine candidate (NCT06891417); however, detailed data on antigen selection, immunogenicity or clinical outcomes have not yet been reported publicly.

Syphilis vaccine development remains at the preclinical stage due to the biological complexity of *T. pallidum* and the need for multivalent antigen formulations. Nonetheless, partial protective immunity observed in animal studies and following human acquisition supports feasibility [20].

*Defining the preferred attributes and use cases* of STI vaccines in advance—what the vaccines should look like and how they should be used—helps ensure that once vaccines are developed, they can be rapidly implemented and meet end‐user needs. WHO has developed preferred product characteristics (PPCs) documents for both gonorrhoea and HSV vaccines [21, 22]. These documents provide strategic guidance on key attributes such as target populations, indications, implementation strategies and desired health impact, helping to ensure that future vaccines align with global public health priorities. WHO vaccine value profiles complement these PPCs by summarizing current evidence on the public health value, the research and development pipeline, and identified data needs to support investment and policy decisions [17, 23].

*Perspective*. The field has made meaningful progress since the STI vaccine roadmap was published. Expanded epidemiologic data and impact modelling now demonstrate the substantial public health value that STI vaccines could deliver. Nonetheless, improved data could strengthen the value proposition.

The global impact of human papillomavirus vaccination demonstrates that effective STI vaccines can be successfully developed, implemented at scale in adolescence, and deliver substantial reductions in transmission and disease outcomes. More recently, the rapid deployment of mpox vaccines during the 2022–2023 global outbreak further illustrates how vaccines can be an effective tool to control sexually associated transmission.

Technological advances have been most notable in gonorrhoea and HSV‐2 research, and the current STI vaccine pipeline remains limited but active. Pipeline attrition reflects the historically low priority given to STI vaccines by industry. Within this landscape, the 4CMenB vaccine represents a valuable stepping stone to accelerate the broader STI vaccine agenda.

STI vaccine development is at a pivotal crossroads. Shifts in global health priorities, research agendas and product development pathways threaten to divert resources. Sustained articulation of public health value, renewed political and scientific commitment, and coordinated investment will be critical to sustaining and expanding the innovation pipeline and translating scientific progress into effective STI vaccines. Ultimately, these vaccines are indispensable for achieving long‐term control of STIs.

## Author Contributions

*Concept development*: RPHP and SLG. *First draft*: RPHP. *Review and revision of draft*: RPHP, SDM, KLS, BG and SLG.

## Conflicts of Interest

The authors do not declare any relevant conflicts of interest.

## Data Availability

This manuscript does not contain original data. Further information can be obtained through the corresponding author.
